# Mitogenome Phylogenetics of Spiruromorpha Porpoise Parasite: Insights Into Phylogeny of *Crassicauda magna*

**DOI:** 10.3390/pathogens14010018

**Published:** 2024-12-30

**Authors:** Lei Han, Yuling Yang, Maolin Lu, Hongyan Yu, Yaxian Lu, Mengchao Zhou, Tianlu Liu, Ruisi Zhang, Bingyao Chen, Zhijun Hou

**Affiliations:** 1 College of Wildlife and Protected Area, Northeast Forestry University, Harbin 150040, China; hanleiwp2022@163.com (L.H.); yangyuling0615@163.com (Y.Y.); 18225391003@163.com (M.L.); yuhongyan2720@163.com (H.Y.); yaxianlu@hotmail.com (Y.L.); zhoumengchao1997@163.com (M.Z.); lexie716@163.com (T.L.); ruisi1103@163.com (R.Z.); 2 College of Life Sciences, Nanjing Normal University, Nanjing 210023, China

**Keywords:** *Crassicauda magna*, mitochondrial genomics, spirurina, marine parasitology

## Abstract

(1) Background: *Crassicauda magna* is a major parasite of large porpoises and whales and has been classified in the Habronematoidea family. However, there has been a great controversy regarding its classification. Mitochondria have an important function in revealing taxonomic and evolutionary history. (2) Methods: In this study, we sequenced the mitochondrial genome of *C. magna* and conducted a phylogenetic analysis with the mitochondrial sequences of species belonging to the Habronematoidea family. (3) Results: The complete mitochondrial genome was 13,604 bp and it has an AT-rich sequence and one non-coding region (NCR). The reconstruction of the ancestral state showed that the gene orders of all species in Spirurina were conserved. The phylogenetic tree indicates that *C. magna* is closer to *Heliconema longissimum* (Physalopteroidea) than *Tetrameres grusi* (Habronematoidea). (4) Conclusions: This study provides new evidence that *C. magna* is phylogenetically closer to Physalopteroidea at the molecular taxonomic level.

## 1. Introduction

In the ocean, parasites are found in a variety of marine organisms, from tiny plankton to large porpoises and whales. In some cases, parasites can hurt or even kill the host by using its resources for their survival. Parasites pose significant threats to marine mammals by causing diseases and impairments that can lead to reduced fitness and, in severe cases, even death; they can also disrupt the ecological balance by affecting the health and population dynamics of these species [1]. The parasites of the class Spiruromorpha are often found in the gastrointestinal tissues of big terrestrial and marine animals [2,3]. Spiruromorpha are a class of parasitic nematodes that alternate their life cycles between vertebrate and invertebrate hosts. There are nine suborders of Spiruromorpha, including Filarioidea, Habronematoidea, and others [4]. The nematode parasite Crassicauda, a genus of the Habronematoidea family, primarily affects huge animals such as porpoises [5]. There are currently 14 morphologically recognized species of Crassicauda, including *C. anthonyi*, *C. bennetti*, *C. boopis*, *C. crassicauda*, *C. fuelleborni*, *C. giliakiana*, *C. grampicola*, *C. magna*, *C. pacifica*, *C. tortilis*, *C. delamureana*, *C. pacifica*, *C. carbonelli*, and *C. duguyi* [6].

The genus of Crassicauda mostly parasitizes the urinary and subcutaneous tissues of cetaceans [7]. Adult or larval *Crassicauda* sp. are found in the kidney or artery walls. They are spread mainly through excreted eggs [8]. Eleven different species of component parasites, including *C. magna*, were found in the stomach, mesentery, and blubber of temperate *Atlantic cetaceans* by Abollo et al. [9]. Abdul et al. presented the basic characterization of *C. magna*’s male tail and forelimbs. It suggests that this species and *C. duguyi* may be synonymous [6]. Thirteen Cuvier’s beaked whales that had been abandoned in the Canary Islands were autopsied in 2016. The adult nematodes discovered in the kidneys were identified morphologically as *Crassicauda* sp. The nucleic acids from two of the kidney worms were most similar to *C. magna*. It has been demonstrated that *Crassicauda* sp. causes severe chronic arteritis in Cuvier’s beaked whales [8]. According to data from Keenan-Bateman et al., *C. magna* mostly affects the pygmy sperm whale’s gill slit gland [7]. In recent years, there have been more parasite reports, which has raised worries about the health of giant cetaceans.

Mitochondria provide energy regulation in life activities. Important biological data may be provided by the mitochondrial genome [10]. The mitochondrial genome is composed of closed-loop molecules with a length of 13.6 to 14.3 kb [11]. The mitochondrial genome is not re-combinable and has experienced rapid evolution. It also has a relatively high matrilineal inheritance rate. The complete mitochondrial genome is becoming an important dataset for resolving taxonomy conflicts because it provides more precise phylogenetic resolution than conventional molecular markers [12]. The mitochondrial genome serves as valuable information for biochemical and molecular biology research, making it a powerful analytical tool for identifying evolutionary relationships and distinguishing closely related species [13].

The mitochondrial genome and its genes play a pivotal role in the taxonomic classification within parasitology data [14,15]. Presently, the classification status of the genus *Crassicauda* primarily relies on morphological characteristics. However, there exists controversy concerning the morphological classification of *C. magna* as belonging to the Habronematoidea family, and the utilization of molecular biology techniques can furnish novel molecular evidence for precisely determining the classification of *C. magna*. In this study, we found an infection of *C. magna* in a stranded and deceased porpoise. To accurately determine the taxonomic status of ascarids using molecular techniques (mitochondrial genes), by extracting DNA and performing next-generation sequencing analysis, we successfully assembled the complete mitochondrial genome of *C. magna* (NCBI:txid1582492, Johnston & Mawson, 1939). By constructing a phylogenetic tree of Spiruromorpha species to identify the taxonomic position of *C. magna*, this contributes to providing a reliable molecular basis for the classification of *Crassicauda* species and presents a new case of parasite infection in marine mammals such as porpoises.

## 2. Materials and Methods

### 2.1. Material and DNA Extraction

The specimens of *C. magna* isolated from Ningbo were obtained from a dead finless porpoise, *Neophocaena sunameri* (NCBI:txid861190, Pilleri and Gihr, 1975), which was stranded in Ningbo, Zhejiang Province, China. A total of eight worms were collected from the neck region of the *N. sunameri*. They were carefully rinsed with PBS buffer (pH = 7.3) and placed in a 75% alcohol solution and kept at −80 °C until the next experiment. The TIANamp Genomic DNA Kit (TIANGEN, Beijing, China) was used to extract the worms’ genomic DNA. Library preparation and sequencing were performed on the DNBSEQ-T1 platform (BGI-Shenzhen, Shenzhen, China).

### 2.2. Mitochondrial Assembly and Annotation Analysis

A total of 80,381,874 reads were obtained through next-generation sequencing. First, the data were trimmed of adapter contamination using NGS-Toolkit [16]. We used two assembly strategies to reconstruct the mitochondrial genome of *Crassicauda* sp.: the NOVOPlasty v4.3.1 [17] and MITObim v1.9.1 programs [18] for mitochondrial the trapping and iterative mapping methods. Both software applications were run using default parameters, specifically: “K-mer = 39, Genome Range = 12,000–20,000, Read Length = 153, Single/Paired = PE, Insert size auto= yes.” The Seed sequence, used as a reference, was downloaded from Genbank and corresponds to the COX1 sequence of *C. magna* (accession number: OQ834322.1). Transfer RNAs (tRNAs), ribosomal RNAs (rRNAs), and protein-coding genes (PCGs) were identified using the online database MITOS (http://mitos.bioinf.uni-leipzig.de/index.py (accessed on 1 February 2023)). The remaining unknown tRNAs were located using the default search mod of ARWEN 1.2 [19]. The codon usage in 12 PCGs was analyzed utilizing the invertebrate mitochondrial genetic code as a reference. The mitochondrial genome was visualized by the CGView online website (http://stothard.afns.ualberta.ca/cgview server/ (accessed on 1 February 2023)). Base composition, codon usage, relative synonymous codon usage (RSCU) values, and nucleotide substitutions were all evaluated using MEGA7.0. The nucleotide composition was calculated using the formulae: AT bias = (A − T)/(A + T); GC bias = (G − C)/(G + C) [20].

### 2.3. Phylogenetic Analysis

To analyze the phylogeny of *C. magna*, the mitotic data for 12 Spiruromorpha and 2 outgroup species (*Ascaris suum* and *Caenorhabditis elegans*) were downloaded from GeneBank (Table 1). One sequence was selected for each species. We performed sequence alignment using ClustalW within the MEGA X software [21] and subsequently trimmed the aligned sequences to remove continuous gap regions. The Bayesian inference (BI) technique was used to build the evolutionary tree. *A. suum* and *C. elegans* were viewed as outgroups while the remainder were classified as in-groups.

PhyloSuite [22] was used to extract 12 PCG sequences from 15 Spiruromorpha mitochondrial genomes, which were aligned and concatenated. The GTR + I + G model was determined to be the best evolutionary model by PartitionFinder2 [23] based on the Akaike information criterion (AIC). The default settings and 1 × 10^6^ metropolitan-linked MCMC generations were utilized for Bayesian inference in MrBayes 3.2.6 [24]. Finally, iTOL (https://itol.embl.de/ (accessed on 1 February 2023)) was applied to edit and visualize the phylogenetic tree.

**Table 1:** Mitochondrial genome information for 13 Spiruromorpha species.

Species	Authorities [25]	Accession No	Mt Genome Size (bp)	AT-Rich	
				Size (bp)	A + T (%)
*Onchocerca ochengi*	Bwangomoi, 1969	NC 031891	13,744	318	83
*Onchocerca* *volvulus*	Leuckart, 1894	NC 056960	13,766	249	82.7
*Brugia malayi*	Brug, 1927	NC_004298	13,657	283	85.2
*Dirofilaria* sp.	-	NC 031365	13,680	279	84.2
*Setaria digitata*	Linstow, 1906	NC 014282	13,839	506	864
*Gongylonema pulchrum*	Molin, 1857	NC 026687	13,798	434	81.1
*Spirocerca lupi*	Rudolphi, 1809	NC 021135	13,780	400	88.5
*Tetrameres grusi*	Shumakovich, 1946	NC 062325	13,709	367	78.2
*Thelazia callipaeda*	Railliet & Henry, 1910	NC 018363	13,668	328	79.6
*C. magna*	Johnston & Mawson, 1939	This study	13,604	338	87
*Heliconema longissimum*	Ortlepp, 1923	NC 016127	13,610	277	96.8
*Physaloptera rara*	Hall & Wigdor, 1918	MH 931178	13,735	518	88
*Camallanus cotti*	Fujita, 1927	NC 036308	17,901	289	74

## 3. Results

### 3.1. The General Features of the Mitogenome of C. magna

**Figure 1:**
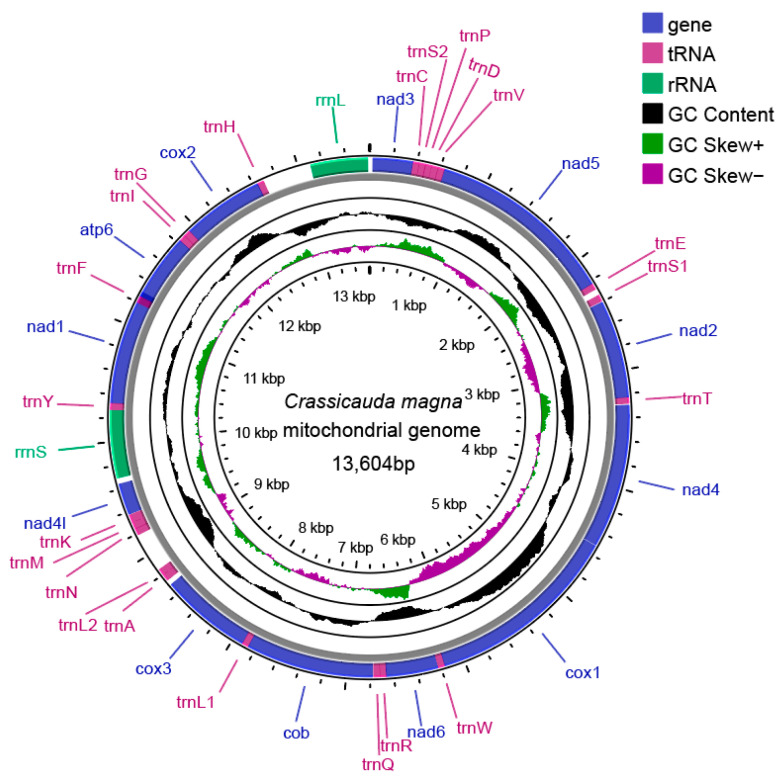
The mitochondrial genome alignment mapping of *C. magna*. PCGs are in purple and blue; rRNAs are in green; tRNAs are in fuchsia. All genes are transcribed in the clockwise direction.

The results indicated that this sequence was 100% identical to a previously reported COX1 sequencing (GenBank accession number MZ222136.1) of *C. magna* from China (Figure S1) [26]. The assembled mitogenome length of *C. magna* was 13,604 bp (GenBank: OQ834322; Figure 1). The genome contains 12 PCGs (nad1, nad2, nad3, nad4, nad5, nad6, COX1, COX2, COX3, nad4L, atp6, and cytb) and 22 transfer RNAs (trnC, trnS2, trnp, trnD, trnV, trnE, trnS1, trnT, trnw, trnR, trnQ, trnL1, trnA, trnL2, trnN, trnM, trnK, trnY, trnF, trnI, trnG, and trnH). The COX1 gene has the greatest coding area (Table 2). All genes are found on the heavy strand of a single DNA strand and are all transcriptionally active. Atpase subunit 8 (atp8) is absent from this mitochondrial genome, which is consistent with the genomic characteristics of other Spiruromorpha species.

### 3.2. Rate of Nucleotide and Codon Usage

The mitochondrial genome nucleotide content of *C. magna* is: A = 22.54%, G = 17.78%, C = 7.26%, T = 54.42%, C + G = 25.04%, and A + T = 74.96% (Table 3). Chain asymmetry is typically represented by AT skewness and GC skewness. The AT skew of *C. magna* is 0.20%, while its GC skew is 0.51%. The findings indicate the *C. magna* mitogenome has an A and T bias in its nucleotide content. The replication of the asymmetric deamination machines in the mitotic genome may be the cause of this bias [27]. However, there is little evidence to prove that invertebrate mitochondrial gene replication is the main cause of strand asymmetry in *C. magna* [28]. The genome’s 12 PCGs accounted for 10,333 bp (75.96%) of the total length. The longest gene was COX1 (1645 bp), while the shortest was nad4L (228 bp). The mitochondrial genome of *C. magna* consists of 18 continuous sections and one overlapping region. The largest size of the spacer area fragment between trnH and rrnL was 416 bp. While the smallest spacer region fragment, of only 2 bp, was found between trnP and trnD. The overlapping region was just 5 bp in size. Among the 12 PCGs, cytb started with ATA, COX2 and COX3 started with TTG, and the other genes started with ATT. There were nine genes with TAA stop codons and three with TAG stop codons: COX1, atp6, and nad4L. It is possible to infer that TAA served as the stop codon for *C. magna*, whereas ATT served as the most typical start codon. The *C. magna* genome has the highest T base content (54.42%), which may be related to the fact that all start and stop codons included T bases (Table 2).

**Figure 2:**
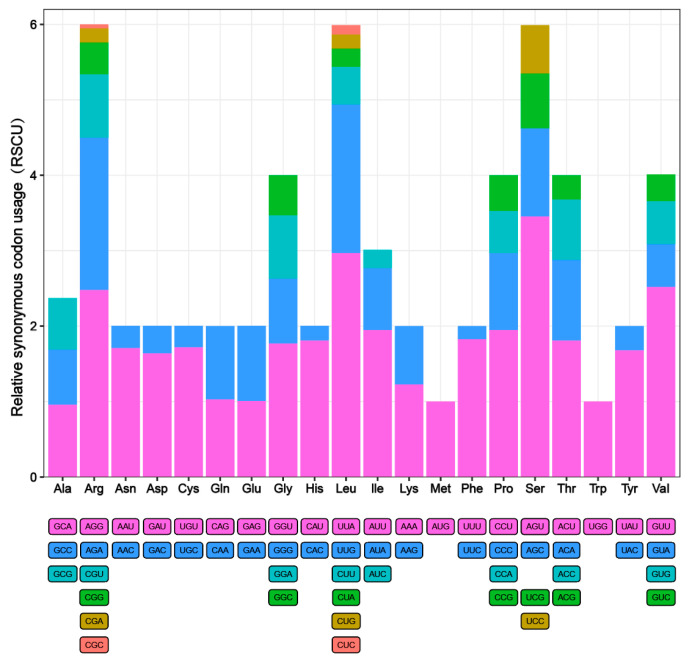
Relative synonymous codon usage (RSCU) of codons. The box below the bar chart represents all codons encoding each amino acid, and the height of the column above represents the sum of all RSCU values.

The RSCU analysis of 12 PCGs showed that the most prevalent amino acids were Leucine (Leu), Phenylalanine (Phe), Isoleucine (IIe), Tyrosine (Tyr), and Valine (Val) (Figure 2). The codons UUA(L), UUG(L), GUU(V), AGA(R), and AGG(R) were the most commonly employed. All of their RSCU levels were larger than 1.9, with UUG having the highest number (L). The fact that every codon has an A or T base indicates the positive AT skew of the PCGs. Synonymous codon deviation is highest in functionally significant gene regions. However, codon deviation is thought to improve translation efficiency and its main correlation with silent site selection [29,30].

### 3.3. The Order of Mitochondrial Genes

**Figure 3:**
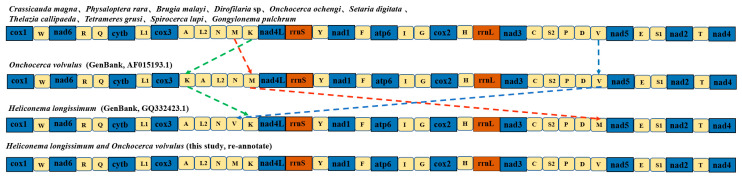
Mitochondrial Gene Arrangement Order in 12 Spiruromorpha Species. The red, green, and blue arrows represent the relative positions of trnM, trnK, and trnV, respectively, across different mitochondrial genome sequences. The frames in blue, orange, and light yellow represent gene, rRNA, and tRNA, respectively.

We downloaded the mitochondrial genomes of 12 species belonging to the class Spirurida and compared the gene orders of *C. magna* with those of 12 other Spiruromorpha species. Spiruromorpha’s gene rearrangement followed the same pattern as the majority of Spiruromorpha. All Spiruromorpha species had the same gene order (COX1, nad6, cytb, COX3, nad4L, nad1, atp6, COX2, nad3, nad5, nad2, and nad4), but not the same order of tRNA genes (Figure 3). *H. longissimum* differed from other species in that the locations of trnV and trnM were switched. *O. volvulus*’s two tRNAs (trnS and trnL) are inconsistent with those of other species (Figure 3). To further validate its reliability, we re-annotated the mitochondrial genomes of *H. longissimum* (GenBank, GQ332423.1) and *O. volvulus* (GenBank, AF015193.1) using GeSeq and MITOS. The results indicated that the discrepancy arose from the annotation of genes in GenBank, and that the mitochondrial gene order within Spiruromorpha species is highly conserved.

### 3.4. Location of AT-Rich and NCR

The mitogenome of *C. magna* contains one non-coding region (NCR), which is between trnH and rrnL (Table 3). The AT-rich is between trnL2-trnN, with a length of 338 bp and an 87% AT content (Table 1). It also shows that *C. magna* is more closely linked to Physalopteroidea than to the Habronematoidea. Because *C. magna*’s AT-rich location is consistent for *H. longissimum* [31].

### 3.5. Phylogenetic Analysis of C. magna

**Figure 4:**
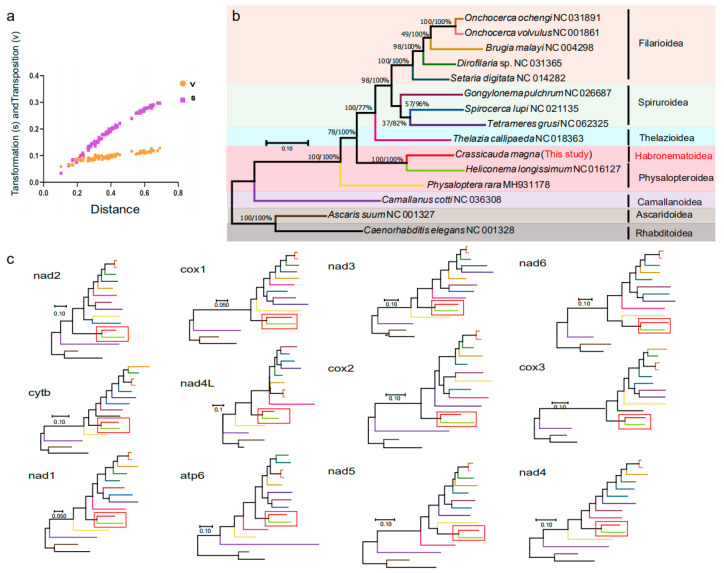
Phylogenetic analysis. (**a**) The transitions (s) and transversions (v) for the complete mitochondrial genome and each protein-coding gene against GTR distance. (**b**) Tandem amino acid sequences of 12 PCGs based on BI analysis, using *Ascaris suum* and *Caenorhabditis elegans* as outgroups, based on the phylogenetic relationships of 13 spirochete species. Posterior probability values are indicated. (**c**) Single-gene phylogenetic analysis of 12 coding genes. The red squares represent the clade of *C. magna* and *H. longissimum*.

DAMBEv7.3.11 was used to determine transformation (s) and transposition (v) in relation to GTR distances. The result is Iss.v < Iss.c, which means that phylogenetic analysis can be performed (Figure 4a). In this study, the concatenated protein sequences of 12 PCGs were employed to predict the evolutionary relationships of *C. magna* in 13 Spiruromorpha species using the BI phylogenetic analysis (Figure 4b). There are two distinct branches on the phylogenetic tree. Filarioidea, Spiruroidea, Habronematoidea, Thelazioidea, and Physalopteroidea combine to one major branch, while Ascaridoidea and Camallanoidea form the other. Despite being members of the Habronematoidea family, *T. grusi* and *C. magna* do not form a single branch. According to the findings of Gao et al., *T. grusi* is more closely related to *G. pulchrum* (Spiruroidea) than to *C. magna* (Habronematoidea) [32]. In the Physalopteroidea family, *C. magna* and *H. longissimum* form an evolutionary branch with a high node support value (BI = 1.00), which is one of the highlights of this phylogenetic tree. The results indicate that *C. magna*’s classification is extremely debatable. To choose the most suitable genetic marker candidates, a single-gene phylogenetic analysis based on 12 coding genes was also carried out (Figure 4c). The phylogenetic tree indicates that *C. magna* and *H. longissimum* cluster together into a clade, suggesting a closer evolutionary relationship.

## 4. Discussion

*C. magna* is a major parasite of large porpoises or whales and has been classified in the Habronematoidea family [5]. However, accurately determining its taxonomic status poses a challenge due to the scarcity of relevant genetic information. Utilizing the structure and conservation of mitochondrial genes offers significant advantages in identifying ancestral relationships [33]. This study employed all known mitochondrial gene sequences of Spiruromorpha to construct a comprehensive phylogenetic tree, which illustrates the phylogeny and divergence times of Spiruromorpha. This significantly expands our understanding of the evolution of Spiruromorpha.

The taxonomy of Spiruromorpha has generated a lot of debate. Following morphological definitions, the subfamily Crassicauda is currently a member of the Tetrameridae family and the Habronematoidea superfamily. Based on the 16S database, it has been concluded that *C. magna* should be classified as a superfamily Acuarioidea rather than the current Habronematoidea [6]. However, this differs from the results of this study. Even though the phylogenetic trees of the other genes are not identical, *C. magna* and *H. longissimum* converge into a single cluster and both have a topological value of 1.0. It also implies that *C. magna* was incorrectly categorized as a member of the Habronematoidea family. In addition, by comparing the conservation of gene order, we also observed that the gene arrangement sequences of *H. longissimum* (GenBank, GQ332423.1) and *O. volvulus* (GenBank, AF015193.1) differ from those of other Spiruromorpha species. Oddly enough, our evolutionary tree analysis revealed that *C. magna* was phylogenetically closer to *H. longissimum*. Yet, the annotation provided by Genbank indicates that the order of two tRNAs (trnM and trnV) in *H. longissimum* differs from that in *C. magna*. Consequently, we re-downloaded the sequence of *H. longissimum* and re-annotated it using the GeSeq and MITOS databases. This re-annotation revealed that the Genbank annotation might be erroneous, as the positions of the two tRNAs (trnM and trnV) in *H. longissimum* are indeed consistent with those in *C. magna*. Therefore, we deem it necessary to clarify this corrected result. This also indirectly corrects a non-existent difference that may have arisen due to issues with database information. Additionally, similar annotation issues were also observed in *O. volvulus* (GenBank, AF015193.1), and we have provided updates and elaborations in Section 3 accordingly. Therefore, we deem it necessary to clarify this corrected result.

New molecular taxonomic evidence for *C. magna* contributes to a more accurate and comprehensive understanding of the diversity of porpoise parasites. The precise identification of *C. magna*’s taxonomic status provides a novel case of its infection in porpoises. Furthermore, these parasite samples were obtained from the necks of porpoises, offering new insights for the clinical diagnosis of parasitic diseases in porpoises and other marine mammals such as whales. By accurately classifying *C. magna*, researchers can gain a deeper understanding of its life cycle, host specificity, and transmission pathways. This knowledge is pivotal for formulating effective conservation strategies aimed at mitigating the parasite’s impact on porpoise and whale populations. In summary, revealing that *C. magna* should be classified as Physalopteroidea rather than Habronematoidea has profound implications for the ecological conservation and biological understanding of porpoises and whales. It provides a more accurate understanding of the parasite’s ecology and biology, enhances conservation efforts, and opens up new research directions that can contribute to the protection and well-being of these marine mammals.

## 5. Conclusions

In the conclusions of this study, we have annotated and comprehensively analyzed the mitochondrial genome of *C. magna* isolated from porpoises, elucidating its composition which includes encoding sequences for 12 proteins, 22 tRNAs, and 2 rRNAs. Additionally, the AT skew within the protein-coding genes (PCGs) was corroborated through an examination of nucleotide content and codon utilization patterns. A phylogenetic analysis was conducted utilizing the mitochondrial genomes of 13 Spiruromorpha species, revealing that *C. magna* exhibits a closer evolutionary relationship to *H. longissimum* (of Physalopteroidea) than to *T. grusi* (also of Habronematoidea).

## Figures and Tables

**Table 2:** Features of the mitogenome of *C. magna*.

Gene	Location	Size (bp)	Anticodon	Start Codon/Stop Codon
*nad3*	26–362	337		ATT/TAA
*trnC*	363–417	55	GCA	
*trnS2*	418–471	54	TGA	
*trnp*	470–523	54	TGG	
*trnD*	525–579	55	GTC	
*trnV*	580–633	54	TAC	
*nad5*	634–2227	1594		ATT/TAA
*trnE*	2228–2282	55	TTC	
*trnS1*	2338–2393	56	TCT	
*nad2*	2403–3230	828		ATT/TAA
*trnT*	3231–3284	54	TGT	
*nad4*	3297–4514	1218		ATT/TAA
*cox1*	4522–6166	1645		ATT/TAG
*trnw*	6167–6222	56	TCA	
*nad6*	6226–6663	438		ATT/TAA
*trnR*	6662–6717	56	ACG	
*trnQ*	6718–6771	54	TTG	
*cytb*	6778–7858	1081		ATA/TAA
*trnL1*	7859–7913	55	TAG	
*cox3*	7914–8690	777		TTG/TAA
*trnA*	8737–8793	57	TGC	
*trnL2*	8796–8850	55	TAA	
*trnN*	9188–9243	56	GTT	
*trnM*	9244–9300	57	CAT	
*trnK*	9303–9369	67	CTT	
*nad4l*	9376–9642	267		ATT/TAG
*rrnS*	9694–10,264	571		
*trnY*	10,270–10,324	55	GTA	
*nad1*	10,325–11,197	873		ATT/TAA
*trnF*	11,200–11,255	56	GAA	
*atp6*	11,250–11,829	580		ATT/TAG
*trnI*	11,830–11,885	56	GAT	
*trnG*	11,886–11,940	55	TCC	
*cox2*	11,941–12,636	696		TTG/TAA
*trnH*	12,635–12,691	57	GTG	
*rrnL*	13,107–13,588	482		

**Table 3:** Nucleotide composition of 12 PCGs, rRNAs, and NCR of *C. magna*. DNA base composition is shown as percentages.

Gene	A (%)	G (%)	T (%)	C (%)	A + T (%)	AT-Skew	G + C (%)
*nad3*	17.04	17.04	58.89	6.67	75.93	−0.55	23.70
*nad5*	19.66	19.84	51.81	8.70	71.47	−0.45	28.53
*nad2*	22.42	14.03	56.95	6.59	79.38	−0.43	20.62
*nad4*	19.83	17.13	55.66	7.37	75.49	−0.47	24.51
*cox1*	21.38	21.44	45.56	11.62	66.39	−0.36	33.07
*nad6*	16.78	14.92	65.60	2.80	82.28	−0.59	17.72
*cytb*	21.90	18.77	50.83	8.50	72.73	−0.40	27.27
*cox3*	22.36	20.08	50.20	7.36	72.56	−0.38	27.44
*nad4l*	15.79	17.98	61.48	4.39	77.63	−0.59	22.37
*rrnS*	30.30	26.26	30.30	13.13	60.61	0.00	39.39
*nad1*	15.70	20.77	56.28	7.25	71.98	−0.56	28.02
*atp6*	19.20	17.57	55.98	7.07	75.18	−0.49	24.64
*cox2*	19.81	23.03	47.99	9.18	67.79	−0.42	32.21
*rrnL*	29.65	17.59	43.72	9.05	73.37	−0.19	26.63
*NCR*	25.78	11.57	58.55	4.10	84.34	−0.39	15.66
*nad3*	17.04	17.04	58.89	6.67	75.93	−0.55	23.70
*nad5*	19.66	19.84	51.81	8.70	71.47	−0.45	28.53
*nad2*	22.42	14.03	56.95	6.59	79.38	−0.43	20.62
*nad4*	19.83	17.13	55.66	7.37	75.49	−0.47	24.51

## Data Availability

The representative nucleotide sequences obtained in this study were submitted to GenBank under the accession number OQ834322.

## Supplementary Materials

The following supporting information can be downloaded at: https://www.mdpi.com/article/10.3390/pathogens14010018/s1, Figure S1: Evolutionary tree of Spirochete species, with *Crassicauda* sp. (MZ222136.1) exhibiting the highest sequence similarity to the COX1 sequence of *C. magna* from this study upon NCBI blast.
