# Supplementary material for: Mitogenome Phylogenetics of Spiruromorpha Porpoise Parasite: Insights Into Phylogeny of Crassicauda magna
Source: Pathogens. 2024 Dec 30;14(1):18. doi: 10.3390/pathogens14010018 (PMC11768356; doi:10.3390/pathogens14010018)
Supplement: Supplementary file 1 [file pathogens-14-00018-s001.zip › pathogens-3364391-supplementary.pdf]

Supplementary Materials

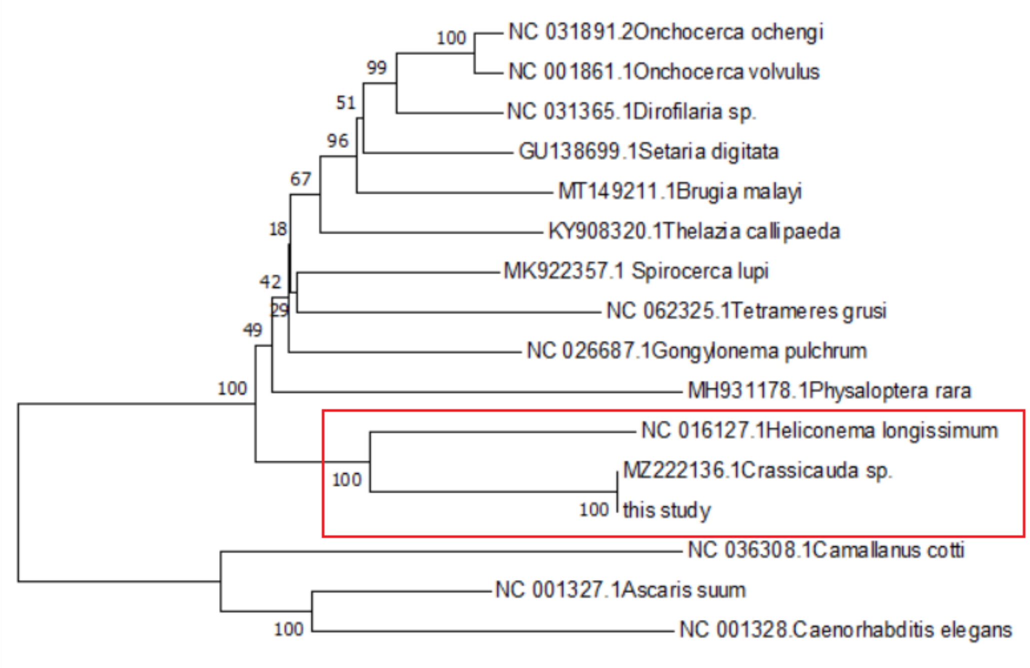

Figure S1. Evolutionary tree of Spirochete species, with *Crassicauda* sp. (MZ222136.1) exhibiting the highest sequence similarity to the COX1 sequence of *C. magna* from this study upon NCBI blast.
